# Enzyme-Based Biosensors and Their Applications

**DOI:** 10.3390/bios13040476

**Published:** 2023-04-14

**Authors:** Yu-Fan Fan, Zhao-Bin Guo, Guang-Bo Ge

**Affiliations:** Shanghai Frontiers Science Center of TCM Chemical Biology, Institute of Interdisciplinary Integrative Medicine Research, Shanghai University of Traditional Chinese Medicine, Shanghai 201203, China; 12022196@shutcm.edu.cn (Y.-F.F.); guozhaobin@shutcm.edu.cn (Z.-B.G.)

Enzymes constitute an extremely important class of biomacromolecules with diverse catalytic functions, which have been validated as key mediators for regulating cellular metabolism and maintaining homeostasis in living organisms [1]. In humans, enzymes play pivotal roles in energy metabolism, metabolite biosynthesis, cellular homeostasis, and the metabolic clearance of various drugs and environmental toxicants (e.g., pollutants, carcinogens, and pesticides) [2]. Given the extreme complexity of the enzyme-mediated biochemical cascade reactions in vivo, enzymes have been proven to be closely involved in numerous pathological processes, including the development of malignant tumors, neurodegenerative diseases, cardiovascular diseases, cerebrovascular diseases, and other forms of organism injury [3]. It has been reported that ~53% of the drugs used to treat these conditions can directly target enzymes (including 30% targeted against kinases), thus validating enzymes’ role as a pivotal class of therapeutic targets [4]. With the rapid development of efficient and high-throughput technologies in the fields of structural biology and life sciences over the past few decades, scientists are racing to develop innovative enzyme-targeting therapies or enzyme-based diagnostic approaches for specific purposes [5]. 

Abnormal changes in the expression or activity levels of enzymes are not only tightly associated with the onset and progression of various diseases (such as cancer, hypertension, and diabetes), but they also significantly affect the outcomes of drugs/toxins in vivo [6,7]. Over the past few decades, an increasing number of enzymes have been identified as pivotal biomarkers and prognostic indicators for various human diseases, which are of tremendous value for disease diagnosis, efficacy assessment, and therapeutic monitoring [8]. For example, alanine transaminase (ALT) has been considered to be a significant liver function indicator in clinical settings. Thus, developing the practical tools for accurately sensing or in situ imaging a target enzyme in real samples is very useful for cell biology, drug screening, individual/species metabolic variations and drug–drug interactions, disease diagnosis, and personalized medicine [7]. Unfortunately, accurately sensing the target enzyme in real samples is not a simple process. Scientists still confront various challenges, including the complexity and diversity of metabolic chains, highly overlapped enzyme substrate spectra, extreme biological matrices, and even the impracticability of analytical devices [6,9,10]. Thus, it is critical to devise more straightforward tools for sensing target enzymes or their related analytes under native physiological and pathological conditions.

Owing to their extraordinary catalytic functions and relatively high substrate specificity, enzymes have been frequently used as key components for the construction of practical, enzyme-based biosensors for the sensing or detection of target analytes in complex biological systems [11]. Over the past few decades, biochemists have devoted exhaustive efforts to developing diverse, enzyme-based biosensors with outstanding specificity, ultra-high sensitivity, and excellent practicability. Components of enzyme-based biosensors have successfully been applied in decoding the dynamic changes of target analytes in real samples or even living systems [12]. Meanwhile, a wide range of innovative concepts and multidisciplinary technologies has been integrated into designing and building innovative and practical enzyme-based biosensors, including electrochemical biosensors and optical biosensors [13]. This Special Issue provides a scholarly platform highlighting recent advances in functional enzyme-based biosensors that greatly facilitate drug discovery, environmental monitoring, food safety, clinical diagnostics, and basic research in medical and biological sciences.

In the field of electrochemical biosensors, scientists frequently utilize oxidoreductases as the critical sensing elements for converting chemical concentrations into electrical signals by catalyzing a target analyte (such as glucose). A polyphenol oxidase (PPO) electrochemical biosensor was proposed to detect and quantify the total phenolic compounds in kombucha samples [14]. The authors successfully optimized the electrochemical detection devices for food quality control and detected phenolic compounds using real food samples under conventional conditions, obtaining a detection limit of 0.13 µM for catechin. Another compact and portable electrochemical biosensor was developed for the analysis of D-2-hydroxyglutaric acid (D2HG) [15]. D-2-hydroxyglutarate dehydrogenase (D2HGDH, a sensing element) and methylene blue (MB, an electron mediator) were immobilized together on a two-dimensional material and then coated onto a gold screen-printed electrode (AuSPE). Upon the addition of D2HG, D2HGDH catalyzed the dehydrogenation of D2HG, while the electrons produced were transferred to the electrode via MB. This sensor displayed a remarkable linear response, with a D2HG concentration over the range of 0.5–120 µM, and successfully detected the levels of D2HG in fetal bovine serum and artificial urine samples.

Recently, optical biosensors have substantially boosted the dynamic visualization of target enzymes due to their facile operation, superior sensitivity, ultra-high spatiotemporal resolution, and isolation- or derivative-free nature. In this Special Issue, Hou et al. designed a highly specific fluorogenic sensor for sensing the hydrolytic activities of human pancreatic lipase (hPL) in complex biological systems [16]. Following the screening of a set of resorufin esters, resorufin lauryl ester (RLE) was identified as the best combination, offering favorable chemical stability, rapid response, excellent specificity, and high sensitivity towards hPL. This optical probe was successfully employed to visualize endogenous PL in living systems with favorable biocompatibility and high imaging resolution. Furthermore, the developed RLE-based high-throughput screening platform efficiently identified several potent hPL inhibitors from among hundreds of clinical drugs and natural products. Yang et al. proposed a microfluidic SlipChip that could be used for the high-throughput screening of hPL inhibitors [17]. As a proof of concept, the practicability and accuracy of this newly developed microfluidic SlipChip were tested using one marketed drug (Orlistat) and two natural hPL inhibitors. Calabria et al. presented a smartphone-based origami microfluidic paper analytical device (µPAD) for quantitatively detecting glucose in blood samples [18]. This method was based on the hydrogen peroxide generated from glucose-oxidase-catalyzed glucose oxidation, which was determined by a luminol/hexacyanoferrate (III) chemiluminescent (CL) system. The µPAD could distinguish hypoglycemic and hyperglycemic blood samples within 20 min. 

Tyrosinase (TYR, E.C. 1.14.18.1) is an initiating and rate-limiting enzyme in melanin biosynthesis responsible for the *ortho*-hydroxylation of L-tyrosine and the oxidation of L-DOPA. It has been implicated as a significant biomarker and therapeutic target for melanoma lesions and skin whitening. Fan et al. comprehensively summarized the substrate preferences, critical structural features, and the enzymatic characteristics and catalytic mechanisms of TYR, particularly emphasizing the recent advances in spectrophotometric assays for sensing TYR activity and their biomedical applications [19]. The methods presented in this review article showed great promise with respect to disease diagnosis and drug discovery. Within this context, Tian et al. first adopted a personal glucose meter (PGM) as a rapid and practicable tool for the analysis of catechol (CA) based on the reduction of the mediator K_3_[Fe(CN)_6_] to K_4_[Fe(CN)_6_] in glucose test strips [20]. CA could be readily oxidized by TYR to *o*-benzoquinone, thereby reducing the residual amount of CA and the PGM readout. In sharp contrast, sodium benzoate (SBA), a TYP inhibitor, could inhibit TYR-mediated CA oxidation. Thus, an inexpensive and simply operable PGM-based method for detecting TYR activity and SBA concentrations was developed based on the TYR-catalyzed reaction. 

Despite the substantial breakthroughs in constructing adaptable biosensors for obtaining insights into various enzyme-induced processes, a host of unresolved challenges still exists with regard to converting such recognition platforms from a research environment to practical applications or industrialization. When constructing electrochemical biosensors, the choice of electrode materials and the immobilization efficiency of enzymes are two essential factors impacting their performance. Furthermore, for optical biosensors, highly specific optical substrates, desirable luminescent materials (e.g., imaging reagents with an appreciable signal-to-noise ratio, high-precision imaging at the subcellular level, or in vivo depth-imaging capacity without any photodamage), sophisticated imaging techniques, and even software upgrades for image reconstruction are required for broader applications in clinical diagnostics. 

In summary, this Special Issue intends to provide an academic platform presenting the recent inroads in innovative enzyme-based biosensors and their applications across a wide range of fields, including medical care, drug discovery, efficacy assessment, environmental monitoring, food safety, and basic research. All the articles published in this collection demonstrate the recent advances in enzyme-based biosensors and their applications, which will be very helpful for future investigations in the biomedical and analytical fields and actively facilitate their applications.
